# Contamination, Source Apportionment and Probabilistic Health Risk of Potentially Toxic Elements in Surface Sediments of the Anning River Basin

**DOI:** 10.3390/toxics14070619

**Published:** 2026-07-15

**Authors:** Wenkai Wang, Pengfei Che, Jinjin Wang, Yue Rao, Jian Luo, Jianbin Chen, Junxi Wang, Yanchang Kun

**Affiliations:** 1 School of Environment and Resource, Xichang University, Xichang 615013, China; 2 Institute of Sedimentary Geology, Chengdu University of Technology, Chengdu 610059, China; 3 College of Earth and Environmental Science, Lanzhou University, Lanzhou 730000, China; 4 School of Information Technology, Xichang University, Xichang 615013, China

**Keywords:** PTEs, source apportionment, PMF, health risk assessment, MCS

## Abstract

The Anning River, traversing the mineral-rich Panxi region, is highly susceptible to contamination by potentially toxic elements (PTEs). This study systematically investigated the contamination profiles, source apportionment, and probabilistic human health risks of eight PTEs in the surface sediments of the basin. Index-based evaluations revealed that Cd acts as the dominant ecological threat, exhibiting extreme enrichment, whereas V, Cr, and Ni reflect natural background signatures. Receptor modeling via Positive Matrix Factorization (PMF) successfully decoupled four distinct sources: mining and smelting emissions (Cd, Zn), natural lithogenic weathering (V, Cr, Ni), mixed traffic/urban inputs (Pb, Cu), and a Tl-specific mixed source. Crucially, while deterministic approaches suggested safe exposure levels, probabilistic Monte Carlo simulations uncovered hidden vulnerabilities: children face a striking 60.51% probability of exceeding the acceptable total carcinogenic risk (TCR) threshold of 1.0 × 10^−4^, primarily governed by Cr and Ni. These findings underscore the urgent need for differentiated environmental management in similar mining-impacted basins. Specifically, stringent source controls for Cd must be implemented alongside exposure pathway interruptions to safeguard vulnerable demographics from Cr and Ni.

## 1. Introduction

With the continuous progression of industrialization and the large-scale exploitation of mineral resources, a significant amount of potentially toxic elements (PTEs) has been released into the atmosphere, water bodies, and soils, becoming a global environmental challenge [1,2,3]. These PTEs are characterized by their persistence and non-biodegradability, which often leads to long-term ecological degradation [4,5,6,7]. Within riverine systems, sediments serve as both a crucial sink and a potential secondary source of PTEs. While sediments can sequester PTEs from the water column through adsorption and co-precipitation, they may also re-release these accumulated elements into the overlying water in response to hydrodynamic disturbances or fluctuations in redox conditions [8,9]. This mobilization not only degrades water quality but also facilitates the bioaccumulation of PTEs in aquatic organisms [10]. Ultimately, these contaminants are transferred through the food chain, posing severe and irreversible risks to human health. Therefore, a comprehensive understanding of the distribution and risk of PTEs in river sediments is essential for environmental management.

Numerous studies have been conducted worldwide to investigate the contamination characteristics of PTEs in river sediments, covering multiple dimensions including spatial distribution patterns, source apportionment, and ecological risk assessment [11,12,13]. Specifically, geochemical indices such as the geo-accumulation index (*I_geo_*), contamination factor (CF), Nemerow’s pollution index (P_N_) and potential ecological risk index (RI) have become standard metrics for quantifying pollution levels and biological threats [14,15,16,17]. Meanwhile, receptor models such as Positive Matrix Factorization (PMF) and multivariate statistical techniques (e.g., PCA) are widely utilized to disentangle the complex contributions of natural and anthropogenic sources [10,18,19,20,21,22]. Furthermore, the Health Risk Assessment model provides a crucial link between environmental concentrations and their potential impacts on local residents through various exposure pathways [21,23,24]. The application of this comprehensive methodological framework to representative riverine systems is essential for regional environmental management and policy-making.

The Anning River, a major tributary of the Yalong River in Southwest China, flows through the Panxi strategic resource innovation zone, which is globally renowned for its abundant vanadium–titanium magnetite reserves [25,26]. As the primary water source for Xichang City and a critical irrigation supply for the surrounding agricultural plains, the environmental integrity of the Anning River is paramount to regional ecological security and sustainable development. However, the unique intersection of intensive mining operations, large-scale smelting activities, and modern intensive agriculture has exerted significant pressure on the riverine environment, potentially leading to the accumulation of PTEs in sediments. Although the Anning River Basin holds important strategic and ecological significance, integrated studies focusing on the pollution characteristics, potential ecological risks, pollution sources, and human health risks of PTEs in its surface sediments remain very limited. Consequently, there is an urgent need to systematically evaluate the current status and risks of PTEs in this specific region to provide a scientific basis for targeted pollution control and environmental management.

Therefore, the primary objectives of this study were to: (1) determine the concentration levels and spatial distribution patterns of PTEs in the surface sediments of the Anning River; (2) evaluate the pollution status and potential ecological risks using various geochemical indices, including the geo-accumulation index (*I_geo_*) and potential ecological risk index (*RI*); (3) differentiate anthropogenic enrichment from natural backgrounds using the enrichment factor (*EF*), and quantitatively apportion the specific source contributions via the Positive Matrix Factorization (*PMF*) model; and (4) estimate the non-carcinogenic and carcinogenic risks to local residents through the health risk model coupled with Monte Carlo simulation (MCS). The findings of this study are expected to provide a comprehensive understanding of the pollution characteristics of potentially toxic elements in the Anning River Basin, and offer crucial scientific support for regional environmental protection and sustainable resource management.

## 2. Materials and Methods

### 2.1. Study Area

**Figure 1:**
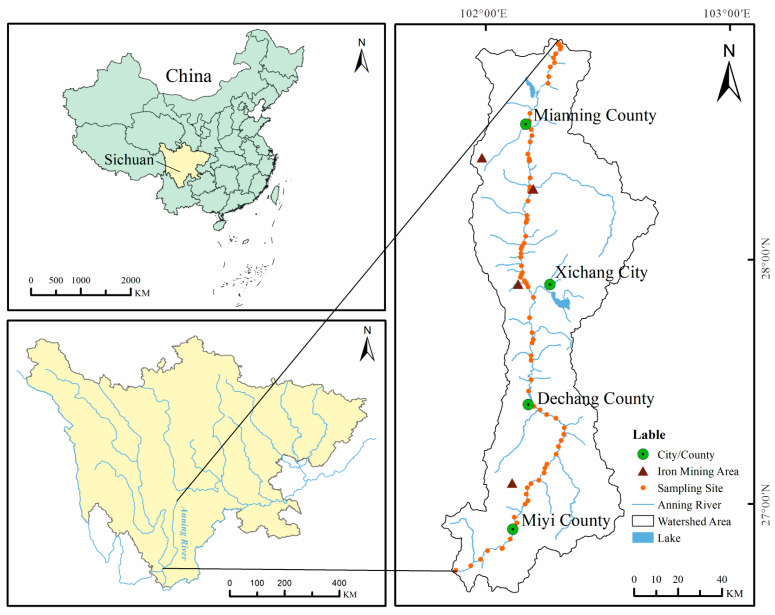
Location of the study area.

The study area encompasses the Anning River Basin, spanning across Xichang and Panzhihua cities in Sichuan Province, Southwest China (Figure 1). As the largest tributary of the Yalong River, the Anning River traverses the Panxi region, with its riverine landscape characterized by a typical valley plain. The region experiences a subtropical plateau monsoon climate [27], with a mean annual temperature of approximately 17.2 °C and an annual precipitation ranging from 1000 to 1100 mm [28]. Geologically, the study area is located within the Panxi Rift Valley, a region globally renowned for its abundant vanadium–titanium magnetite reserves [29]. While intensive agricultural activities and rapid urbanization are concentrated across the valley plain, the regional economy is predominantly driven by mining, ore processing, and smelting industries situated in the upper and middle reaches of the basin.

### 2.2. Sample Collection

During January to February 2024, a total of 80 surface sediment samples (0–10 cm depth) were collected along the Anning River. To ensure a comprehensive characterization of PTE distribution, the sampling sites were systematically distributed from north to south. At each sampling site, where the average flow velocity was 0.4 ± 0.1 m/s, a composite sample was formed by mixing three sub-samples collected within a 3 m radius using a stainless-steel grab sampler to ensure spatial representativeness. The precise geographic coordinates of each location were recorded using a GPS. All collected samples were sealed in clean, pre-labeled polyethylene bags, stored in portable coolers at 4 °C, and immediately transported to the laboratory. In the laboratory, the samples were air-dried at room temperature in a clean environment. After removing large gravel and organic debris, the dried sediments were ground using an agate mortar and passed through a 200-mesh nylon sieve. The processed samples were subsequently stored in a desiccator until chemical analysis.

### 2.3. Chemical Analysis and Quality Control

The total concentrations of PTEs (e.g., V, Cr, Ni, Cu, Zn, Cd, Pb and Tl) in the sediment samples were determined using inductively coupled plasma mass spectrometry (ICP-MS; NexION^®^ 2000, PerkinElmer Inc., Waltham, MA, USA). The sample digestion procedure was conducted following the method described by Wang et al. [30] with minor modifications. Briefly, approximately 0.1000 g of each powdered sediment sample was accurately weighed into a Teflon digestion vessel. A mixture of 1 mL HNO_3_ and 1 mL HF was added to the vessel. The digestion process was carried out in an oven with a programmed temperature sequence of 100 °C and 180 °C. To remove residual acids, the mixture underwent two stages of acid evaporation to near dryness. Subsequently, the residue was heated at 140 °C and then diluted to a final volume of 10 mL with ultra-pure water for ICP-MS analysis.

Stringent quality assurance and quality control (QA/QC) protocols were implemented throughout the analytical process. These included the analysis of reagent blanks, duplicate samples (10% of the total), a certified reference material (GSS-4a, provided by the Institute of Geophysical and Geochemical Exploration, Chinese Academy of Geological Sciences, IGGE, Beijing, China), and a certified standard solution (GNM-M220198-2013, provided by the National Analysis Center for Nonferrous Metals and Electronic Materials, NACNAM, Beijing, China). The recovery rates for the analyzed PTEs ranged from 87.2% to 109.4%, and the relative standard deviation (RSD) of the duplicate samples was consistently below 5%. All laboratory glassware and digestion vessels were pre-soaked in 10% HNO_3_ for 24 h and rinsed with deionized water to prevent potential cross-contamination.

### 2.4. Statistical and Data Analysis

Descriptive statistics (including the mean, minimum, maximum, and standard deviation) for PTE concentrations were calculated using Minitab (version 22.0; Minitab^®^ LCC., State College, PA, USA). Correlation analysis and other multivariate statistical tests were also conducted using Minitab to explore the interrelationships among the selected elements. The geographic map of the sampling sites and the spatial distribution patterns of the PTEs were generated using ArcGIS (version 10.8; Environmental Systems Research Institute, Inc., Redlands, CA, USA). All graphical representations were produced using Origin Pro (version 2022; OriginLab Corp., Northampton, MA, USA).

### 2.5. Assessment Methods

In the present study, a comprehensive multi-model framework was employed to systematically evaluate the environmental impacts of PTEs in the surface sediments of the Anning River Basin. Specifically, the *I_geo_* was utilized to quantify the pollution levels of individual PTEs, while the *RI* was applied to evaluate the associated ecological threats to the aquatic ecosystem. To identify and quantitatively apportion the origins of these contaminants, the *EF* and the *PMF* model were utilized conjunctively. Furthermore, the health risk model was conducted to estimate the potential adverse health effects (both non-carcinogenic and carcinogenic) posed to local populations through various exposure pathways to the sedimentary PTEs. The detailed calculation formulas, parameters, and grading criteria for all aforementioned assessment models are systematically summarized in Table 1.

## 3. Results and Discussion

### 3.1. PTE Characteristics in River Sediments

The descriptive statistics of potentially toxic elements (PTEs) in the surface sediments of the study area are summarized in Table 2. The mean concentrations of the analyzed elements decreased in the following order: Zn (168.95 ± 108.28 mg/kg) > Cr (135.29 ± 168.19 mg/kg) > V (117.32 ± 37.96 mg/kg) > Pb (116.67 ± 156.97 mg/kg) > Ni (45.23 ± 50.01 mg/kg) > Cu (44.96 ± 17.88 mg/kg) > Cd (0.85 ± 0.80 mg/kg) > Tl (0.69 ± 0.17 mg/kg). The results revealed that the average concentrations of all eight PTEs exceeded their respective background levels, indicating widespread accumulation of potentially toxic elements across the study area. National stream sediment background values were used for all PTEs to match the study medium, except for Tl. As Tl is not covered in China’s public national stream sediment geochemical baseline datasets, the authoritative Sichuan provincial soil Tl background value was adopted. This choice is justified by the strong geochemical homology between catchment soils and fluvial sediments, making the regional soil value the most reliable available proxy for local natural Tl baselines. Most notably, the mean concentrations of Cd and Pb were substantially elevated, reaching approximately 7.73 and 5.30 times their background values, respectively. Such pronounced enrichment strongly suggests the dominant influence of intense anthropogenic inputs, including industrial discharges, mining activities, and urban runoff. Other elements, including Zn, Cr, Cu, and Ni, exhibited moderate enrichment, with mean concentrations factors ranging from 2.06 to 2.60 times their background values. In contrast, V and Tl displayed the lowest mean concentrations factors (1.56 and 1.25 times, respectively), implying a relatively stronger association with the natural geological baseline rather than anthropogenic disturbances.

The coefficient of variation (CV) serves as a robust statistical indicator for evaluating spatial dispersion and distinguishing naturally occurring elements from those subjected to anthropogenic disturbances. Generally, a CV exceeding 50% is indicative of high spatial variability, suggesting that the element is highly susceptible to human activities and likely originates from discrete point sources [35]. In this study, Pb (134.54%), Cr (124.31%), Ni (110.56%), Cd (93.94%), and Zn (64.09%) all demonstrated markedly elevated CV values (>50%), reflecting a heterogeneous spatial distribution across the sampling sites. Such high variability is typically driven by localized anthropogenic inputs, such as mining operations, smelting activities, industrial effluents, or traffic-related emissions. In contrast, Cu (39.77%), V (32.36%), and Tl (23.87%) exhibited relatively lower CV values (<50%), suggesting a more homogeneous spatial distribution. These elements are more likely governed by natural pedogenic processes, lithological inheritance, or diffuse non-point sources, with comparatively limited anthropogenic influence. The pronounced anthropogenic influence on high-CV elements is further corroborated by a systematic comparison of their mean and median values.

In comparison with other domestic rivers, the concentrations of Pb, Cd, and Zn in the study area were substantially elevated relative to those documented for the Jinsha River (22.23, 0.25, and 56.50 mg/kg), Weihe River (21.23, 0.41, and 67.90 mg/kg), and Danjiang River (26.00, 0.49, and 91.99 mg/kg). Specifically, the Pb concentration in this study are consistently higher than those in other study areas. This marked difference suggests that the local enrichment pattern of lead is driven by region-specific anthropogenic activities. Although the concentrations of Cr, Cu, and Zn were higher than those in most studied Chinese rivers, they remained considerably lower than the values reported for the Yellow River. This pattern is attributable to the Yellow River’s exceptionally high suspended sediment load and cumulative multi-source pollutant inputs [36,37]. From an international perspective, the contamination levels in the study area were markedly more severe than those in the Bhairab–Rupsha Rivers of Bangladesh, where concentrations of Cr, Ni, and Pb were notably low. Furthermore, when compared to other major international rivers, the concentrations of Cr, Ni, and Zn in the current study were approximately twice as high as those reported for the Ganga River, India, while Cu levels remained comparable. Similarly, relative to the Nile River, the study area exhibited significantly higher levels of Cr, Cu, Cd, and Pb, though the Zn concentration was an exception, being comparatively lower in our study than in the Nile. This substantial regional disparity underscores the considerably greater degree of anthropogenic pressure exerted on the current study area relative to less industrialized river basins. Overall, the comparative analysis reveals that while the study area exhibits moderate to high enrichment of most PTEs, the levels of Pb and Cd are particularly concerning, consistent with those typically observed in basins experiencing intensive industrial and mining perturbations.

The observed differences in metal concentrations across river basins reflect a combination of geological, anthropogenic, and methodological factors. Geological background varies substantially among regions, and rivers draining mineralized zones such as the Panxi area naturally have higher baseline metal concentrations than watersheds with non-mineralized bedrock. The type and intensity of human activities also differ markedly, with mining-dominated rivers like the Anning showing a distinct Cd-Zn pollution signature, in contrast to industrial urban rivers where Pb and Cu are typically more prominent. Methodological differences in sampling and analytical procedures further complicate direct comparisons across studies. Overall, this study provides valuable data from the Panxi mining region, an area that has received relatively less attention in the international literature compared to mining districts in other parts of the world.

**Table 2:** Levels of PTEs in sediments from the Anning River and those from other relevant studies (mg/kg).

	V	Cr	Ni	Cu	Zn	Cd	Pb	Tl	References
Min-Max	53.41–282.10	32.68–1458.02	7.40–454.63	11.70–129.86	64.42–919.99	0.17–5.39	23.95–974.55	0.45–1.56	This study
Average ± SD	117.32 ± 37.96	135.29 ± 168.19	45.23 ± 50.01	44.96 ± 17.88	168.95 ± 108.28	0.85 ± 0.80	116.67 ± 156.97	0.69 ± 0.17	
Median	109.29	99.46	38.62	44.05	152.24	0.68	69.34	0.65	
Coefficient of variation (%)	32.36	124.31	110.56	39.77	64.09	93.94	134.54	23.87	
Background values of sediments in China	75	54	22	20	65	0.11	22	0.55 *	[38,39]
Jinsha River, China	-	161.29	41.25	58.57	56.50	0.25	22.23	-	[40]
Weihe River, China	-	64.20	29.93	23.00	67.90	0.41	21.23	-	[4]
Yellow River, China	-	216.89	61.68	247.55	332.51	0.90	-	-	[41]
Heilongjiang, China	-	42.4	-	11.2	62.5	0.13	28.16	-	[42]
Danjiang River, China	-	83.94	31.74	31.12	91.99	0.49	26.00	-	[43]
Bhairab and Rupsha Rivers, Bangladesh	-	4.64	24.98	26.77	-	0.08	4.15	-	[44]
Ganga River, India	-	63.8	24.6	47	72.3	-	-	-	[45]
Nile River, Egypt	-	29.40	-	16.20	207.0	0.31	4.32	-	[46]

* It is the soil background value of Sichuan Province [39].

### 3.2. The Spatial Distribution of PTEs in River Sediment

**Figure 2:**
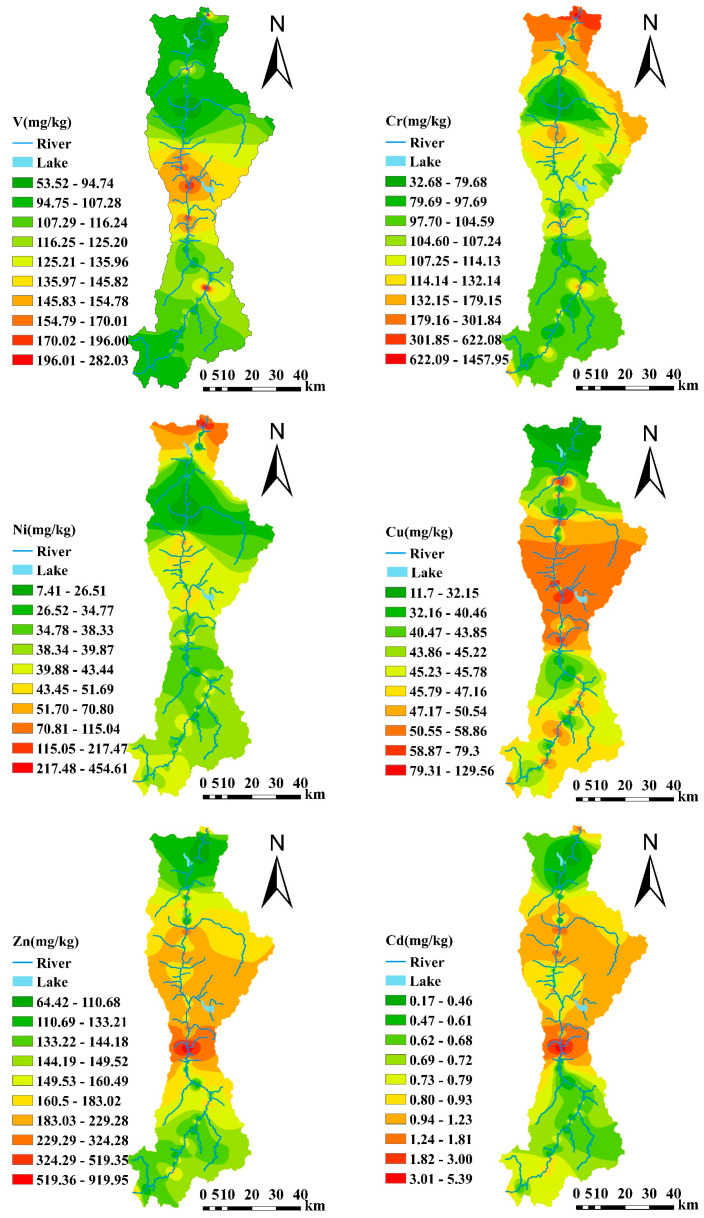

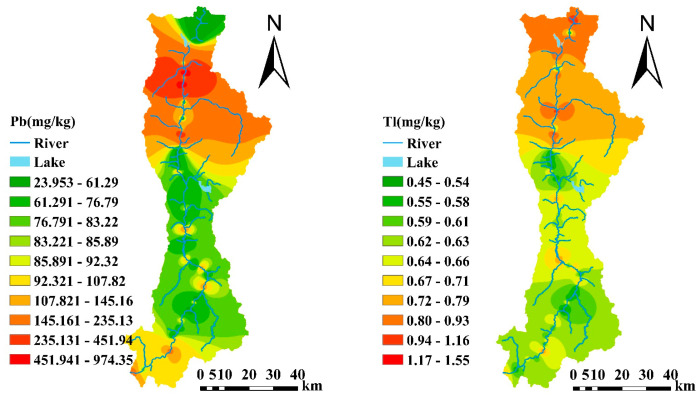
Spatial distribution map of PTEs.

The spatial distributions of eight PTEs in the sediments of the Anning River are illustrated in Figure 2. Overall, the elements exhibit pronounced spatial heterogeneity, with the high- and low-concentration zones varying considerably among different elements. Notably, Cr and Ni exhibited similar spatial patterns, as did the group comprising Zn, Cu, and Cd. This spatial coupling suggests that these elements may share similar sources and undergo comparable environmental migration and transformation processes within the study area.

Spatially, significant high-concentration zones for Cr and Ni were observed in the upstream sediments of the Anning River. Their enrichment levels in this area were substantially higher than in other reaches, reflecting a localized, high-intensity accumulation. In contrast, the high-concentration zones of Zn, Cu, and Cd were predominantly confined to the middle reach, an area characterized by extensive industrial land use, including iron mining operations and metal smelting facilities. Consequently, industrial activities and mineral resource exploitation are considered the key anthropogenic drivers of elemental enrichment in this specific section of the river [31,47].

The high-concentration hotspots of Pb are predominantly concentrated in the upstream reach of the northern watershed, forming an extensive and spatially continuous enrichment belt with markedly elevated peak values at localized centers. This highly clustered hotspot pattern is characteristic of point-source pollution, strongly implying the presence of intensive Pb-related industrial activities in the upstream area, such as lead–zinc mining and metal smelting operations [32]. As the river flows from north to south, Pb concentrations undergo a pronounced stepwise decline through the middle reach, reflecting the progressive dilution and gravitational settling of PTEs during downstream fluvial transport. By the time the river enters the middle-to-lower reach, Pb concentrations have broadly decreased to relatively low levels, with only sporadic and weakly enriched patches persisting along the southern periphery of the watershed. Overall, intense anthropogenic point-source inputs in the upstream area represent the dominant factor governing the spatial distribution pattern of Pb throughout the watershed.

### 3.3. Pollution and Risk Assessment of PTEs

#### 3.3.1. EF Characteristics in River Sediments

**Figure 3:**
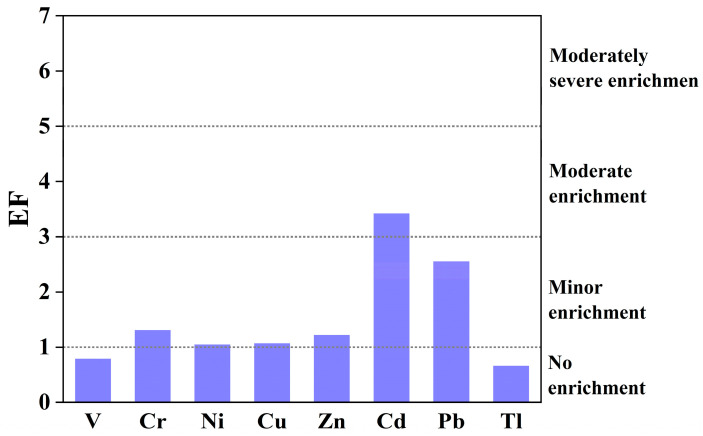
The EF of in the surface sediments of the Anning River.

The EF was employed to evaluate the degree of anthropogenic influence on PTE accumulation in the surface sediments, with the results illustrated in Figure 3. The mean EF values followed the order of Cd > Pb > Cr > Zn > Cu ≈ Ni > V > Tl. Among the analyzed elements, Pb, Cr, and Zn showed EF values between 1 and 3, suggesting minor enrichment, whereas Ni and Cu were close to 1, indicating only slight or negligible enrichment. In contrast, the EF values of V and Tl were lower than 1, reflecting no enrichment.

Overall, the EF results identify Cd as the most substantially enriched element in the study area, making it the primary indicator of anthropogenic perturbations. The moderate enrichment of Cd indicates substantial external inputs beyond the natural geochemical background. Pb also showed a relatively high EF value (EF = 2.55), indicating minor enrichment and suggesting a certain degree of anthropogenic contribution. Cr and Zn displayed slight enrichment, implying that they may be affected by mixed sources, including both natural background contributions and human activities. By contrast, the EF values of V and Tl were lower than 1, suggesting that these elements were not significantly enriched and were mainly controlled by lithogenic sources. In particular, although V showed a relatively elevated concentration, its low EF value indicates that its occurrence is more closely intrinsically linked to the regional geological background (e.g., vanadium–titanium magnetite weathering) rather than recent anthropogenic discharges.

#### 3.3.2. The *I_geo_* Characteristics of River Sediments

**Figure 4:**
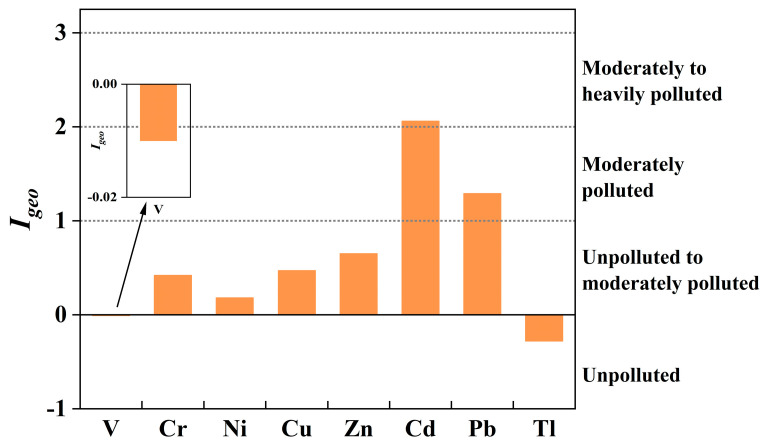
The *I_geo_* of PTEs in the surface sediments of the Anning River. The arrow in the figure points to an enlarged view of the *I_geo_* value for V.

The geo-accumulation index (*I_geo_*) was employed to evaluate the contamination degree of PTEs in the surface sediments of the Anning River, with the results illustrated in Figure 4. The mean of *I_geo_* values followed a descending order of Cd (2.06) > Pb (1.29) > Zn (0.65) > Cu (0.47) > Cr (0.42) > Ni (0.18) > V (−0.01) > Tl (−0.28). Among the analyzed elements, Cd showed the highest *I_geo_*, slightly exceeding 2, corresponding to moderate to heavy pollution. In contrast, Zn, Cu, Cr, and Ni all had *I_geo_* values between 0 and 1, falling into the category of unpolluted to moderately polluted. Overall, the *I_geo_* results reveal that Cd and Pb are the dominant pollution contributors in the study area, whereas the other elements show relatively low to moderate accumulation. The *I_geo_* value of Cd is particularly high, with 91.3% of the sampling points exceeding 1, which is consistent with the high degree of enrichment and high coefficient of variation in Cd relative to the background value, indicating that Cd is significantly influenced by anthropogenic activities. Considering the regional characteristics of the Anning River basin, the elevated Cd level is likely associated with mining, smelting, and related industrial activities [48]. Similarly, the moderate accumulation of Pb further suggests the influence of ore exploitation, metallurgical emissions, and possibly traffic-related inputs. This pattern is consistent with the regional mining and metallurgical background. Smelting-derived emissions, particularly metal-laden particulates, can undergo atmospheric transport before being introduced into riverine systems via wet deposition, thereby exacerbating the accumulation of PTEs (such as Cd and Pb) in the sediments [49]. It is worth noting that Tl, despite showing low enrichment levels, deserves attention due to its high intrinsic toxicity [50], even though its concentrations are only slightly above background values.

#### 3.3.3. Probabilistic Assessment of *I_geo_* Based on Monte Carlo Simulation

**Figure 5:**
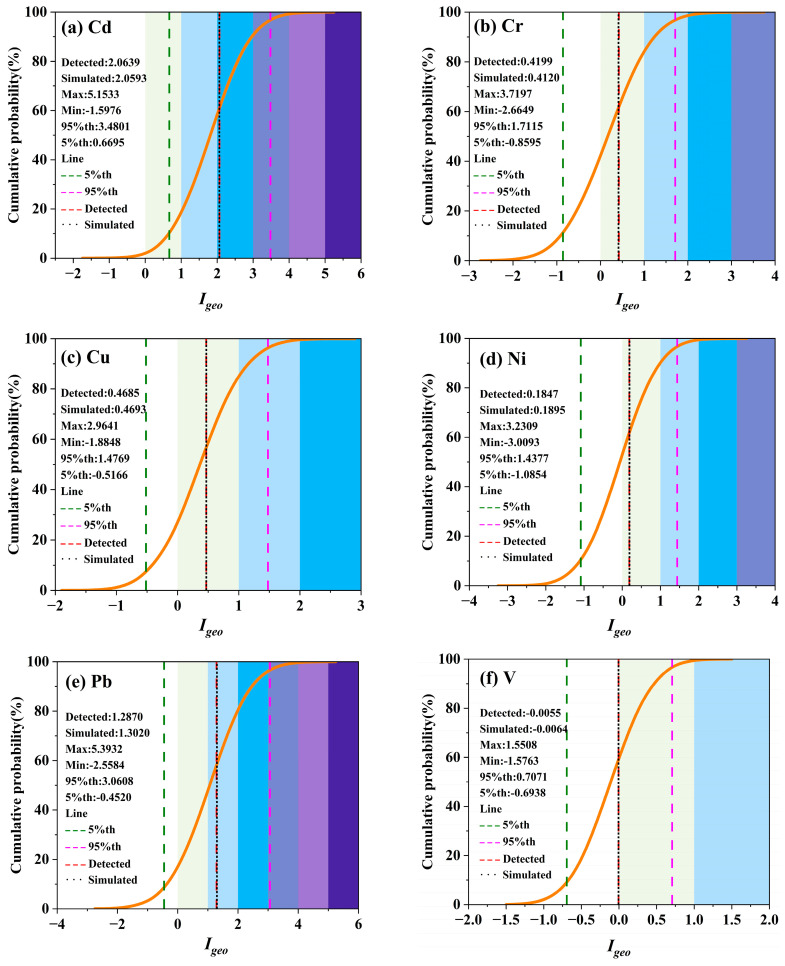

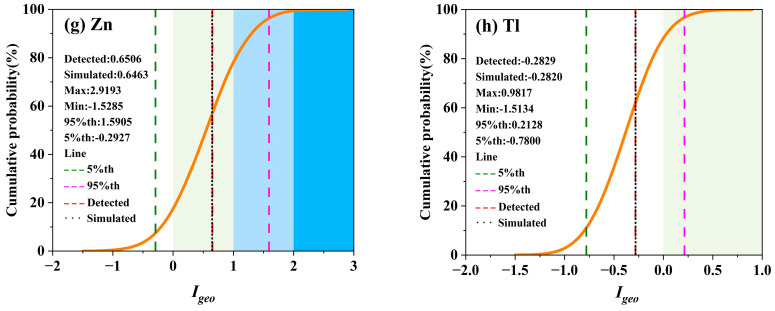
Monte Carlo simulation results of geo-accumulation index (*I_geo_*) for eight potentially toxic elements in the surface sediments of the Anning River.

To quantify the uncertainty associated with the *I_geo_* and to overcome the limitations of deterministic evaluation based solely on average values, a Monte Carlo simulation (MCS) was performed for the eight PTEs (Figure 5). The results demonstrated exceptional concordance between the deterministic and simulated *I_geo_* values. For instance, the detected/simulated values for Cd (2.0639/2.0593), Pb (1.2870/1.3020), V (−0.0055/−0.0064), and Tl (−0.2829/−0.2820) were highly consistent, confirming that the probabilistic model reliably reproduced the contamination profiles of the surface sediments.

Among all elements, Cd exhibited the most pronounced probabilistic contamination pattern. The simulated mean *I_geo_* value of Cd was 2.0593, with the 5th and 95th percentiles being 0.6695 and 3.4801, respectively. This indicates that Cd remained above the unpolluted threshold in almost all simulated scenarios and had a substantial probability of reaching *I_geo_* > 2, corresponding to a moderately to heavily polluted level. Therefore, Cd contamination is not merely driven by a few extreme observations, but rather represents a robust and persistent pollution feature across the study area. Pb showed the second-highest probabilistic contamination level. Its simulated mean *I_geo_* value was 1.3020, with the 5th percentile of −0.4520 and the 95th percentile of 3.0698. This wide interval suggests that Pb can range from unpolluted to moderately or even moderately to heavily polluted under different scenarios, indicating strong anthropogenic influence accompanied by pronounced spatial heterogeneity.

In contrast, Cr, Cu, Ni, and Zn showed simulated mean *I_geo_* values between 0 and 1, indicating generally unpolluted to moderately polluted conditions. Their 5th percentile values were all below zero, whereas their 95th percentile values exceeded 1, implying that these elements spanned multiple pollution classes under uncertainty conditions. This pattern suggests that their accumulation was jointly controlled by natural background variability and localized anthropogenic inputs. Among them, Zn exhibited a relatively higher simulated mean and upper percentile, implying a comparatively stronger anthropogenic contribution. V and Tl displayed the lowest probabilistic contamination levels. Their simulated mean *I_geo_* values were −0.0064 and −0.2820, respectively, and even their 95th percentile values remained below 1, indicating that they were generally within the unpolluted range and mainly controlled by lithogenic sources.

Overall, the MCS results were highly consistent with the deterministic *I_geo_* assessment and further confirmed that Cd and Pb were the major pollution contributors in the study area, with Cr, Cu, Ni, and Zn exhibited mild but uncertain accumulation, and V and Tl were generally unpolluted.

#### 3.3.4. The Ecological Risk of River Sediments

**Figure 6:**
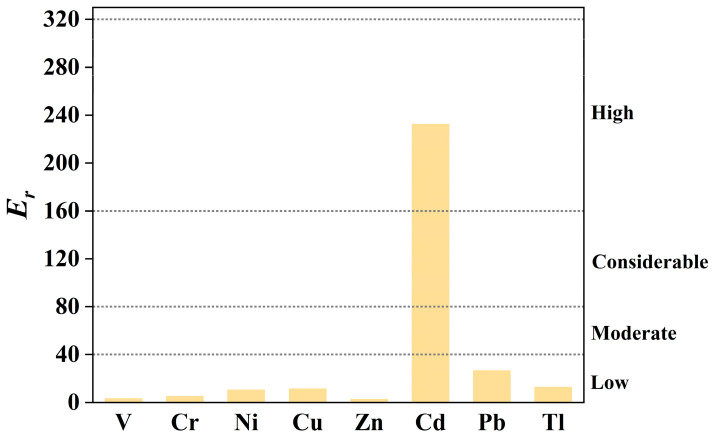
The potential ecological risks of different PTEs in river sediments.

The potential ecological risk coefficients (*E_r_*) of the analyzed elements are shown in Figure 6. The *E_r_* values followed the descending order of Cd > Pb > Tl > Cu > Ni > Cr > V > Zn. With the exception of Cd, all PTEs exhibited Er values below 40. This result indicates that Cd is the dominant ecological risk contributor in the surface sediments of the Anning River. The extremely high ecological risk posed by Cd is attributable to both its significant enrichment relative to the background and its high toxic-response coefficient. To comprehensively evaluate the combined ecological threat posed by multiple PTEs, the comprehensive potential ecological risk index (RI) was calculated. The average RI value for the surface sediments of the Anning River was determined to be 303.64. Based on established grading criteria (200 ≤ RI < 400), this value indicates that the study area as a whole is subjected to a considerable ecological risk [34]. It is crucial to highlight the disproportionate contribution of individual elements to the overall RI. Although seven out of the eight analyzed PTEs (Pb, Tl, Cu, Ni, Cr, V, and Zn) fell into the low-risk category, the total RI still reached a considerable level. This elevated overall risk is almost entirely driven by Cd, which alone contributed approximately 76.5% (*E_r_* = 232.25) to the total RI.

The pronounced Cd-dominated risk pattern provides evidence for point-source anthropogenic contamination rather than natural background variation or diffuse pollution. This interpretation is further corroborated by the *I_geo_* and EF results, which consistently identify Cd as the most severely anthropogenically influenced pollutant among the studied elements. In the Panxi region—one of China’s most important non-ferrous metal mining and smelting bases—Cd contamination in river sediments can be predominantly attributed to long-term mining, beneficiation, and smelting activities. As a typical associated element of polymetallic ores (e.g., Pb-Zn and V-Ti magnetite ores), Cd is readily released during ore processing and enters the aquatic environment through two primary pathways: (1) leachate from tailings piles and waste rock dumps transports dissolved and particulate Cd into surface runoff, ultimately accumulating in river sediments; and (2) Cd-bearing particulate matter from industrial flue gas emissions enters the water body via atmospheric dry and wet deposition. The combined effect of these inputs, accumulated over decades of mineral exploitation, has shaped the current Cd-dominated ecological risk profile of the Anning River sediments.

Consequently, Cd should be unequivocally identified as the priority pollutant for future environmental monitoring, pollution control, and sediment remediation strategies in the Anning River basin, with particular attention to mining and smelting sources.

### 3.4. Source Apportionment

**Figure 7:**
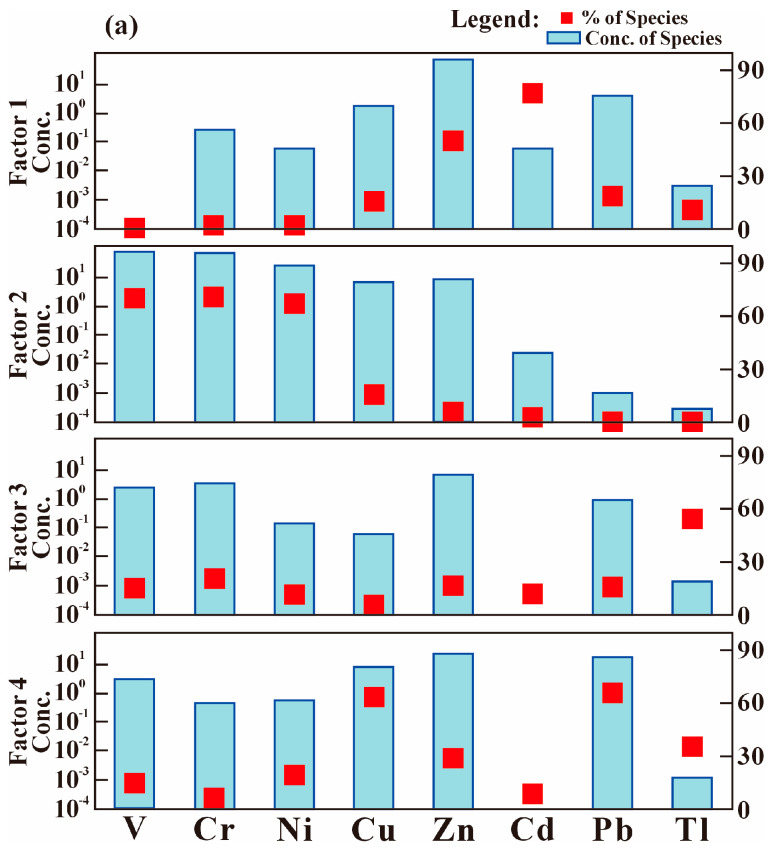

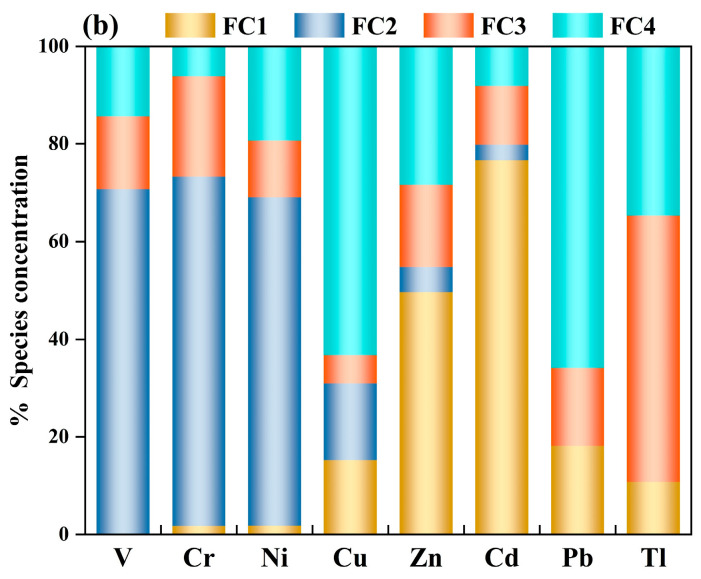
Base factor profiles of potentially toxic elements in the surface sediments of the Anning River obtained from PMF: (**a**) Results of the PMF factor analysis, (**b**) Relative contributions of the factors to individual heavy metals.

Model performance and factor solution robustness were verified with standard PMF diagnostic metrics; full details are provided in Supplementary Materials Text S1. Specifically, the near-unity Q_robust_/Q_true_ ratio confirmed negligible outlier influence on model fitting, and >95% of species residuals fell within the −3 to 3 range, indicating no systematic prediction bias. The 4-factor solution was selected as optimal: increasing factor counts beyond 4 produced chemically uninterpretable mixed/split factors, while reducing to 3 factors merged geochemically distinct sources. Using this validated model, four distinct source factors were resolved for potentially toxic elements (PTEs) in surface sediments of the Anning River (Figure 7).

Factor 1 was characterized by high contributions to Cd (76.6%) and substantial contribution to Zn (49.7%), forming a typical Cd-Zn association. Since Cd commonly occurs as an associated element in Zn-bearing ores, both elements can potentially be simultaneously released during mining, ore beneficiation, smelting, and tailing weathering [51,52]. On this basis, Factor 1 was interpreted as a mining- and smelting-related anthropogenic source. This interpretation is consistent with the pollution assessment results, which identified Cd as the most enriched and ecologically hazardous element in the study area.

Factor 2 is most likely associated with V, Cr, and Ni, contributing more than approximately 67.3–71.5% to these elements. This V-Cr-Ni assemblage is consistent with the regional geological background and suggests a lithogenic control. Combined with the low EF and relatively low *I_geo_* values of these elements, Factor 2 was identified as a natural geogenic source, mainly related to parent material weathering, soil erosion, and sediment transport processes [53,54,55].

Factor 3 was primarily characterized by Tl, indicating a relatively independent source pattern for this element. It should be noted that factors dominated by a single element generally carry greater interpretive uncertainty in PMF analysis, as they provide fewer elemental associations to constrain source identification. The separation of Tl into an independent factor may reflect its unique geochemical behavior, which differs from that of the other studied metals. Based on the available evidence, this factor may represent a Tl-specific source mainly associated with local geological background, with possible minor anthropogenic or localized enrichment influences. Tl can naturally occur in parent rocks, soils, K-bearing minerals, clay-rich materials, and Tl-bearing sulfides such as pyrite [56,57,58]. This interpretation is supported by the low *EF* and *I_geo_* values of Tl, which suggest limited anthropogenic enrichment; however, the slight exceedance of the regional background value indicates that minor external inputs or local enrichment cannot be fully excluded [59]. Therefore, this interpretation remains tentative due to the single-element nature of the factor. Additional geological or geochemical evidence would be needed to more definitively characterize the nature of this source. Although Tl did not register as a high-level pollutant in the index-based assessments, its extreme inherent toxicity means that this source warrants continued environmental monitoring, even at low concentrations.

Factor 4 was mainly associated with Cu and Pb, accounting for about 63.2% and 65.8% of their respective contributions, and also showed moderate inputs to Tl, Zn, and Ni. This Cu-Pb association appears to be related to mixed traffic, industrial, and urban sources. Cu is commonly linked to machinery abrasion, industrial effluents, and urban runoff [60,61], whereas Pb may originate from historical traffic emissions, road dust, and industrial atmospheric deposition [62,63]. Crucially, the separation of Pb from the Cd-Zn factor (F1) implies that Pb in the Anning River is not primarily derived from the same localized mining activities, but rather stems from broader, diffuse anthropogenic inputs associated with urbanization and transportation.

Overall, the PMF analysis suggests that the PTEs in the study area are jointly controlled by natural geological weathering (V, Cr, Ni), mining and smelting activities (Cd, Zn), mixed traffic and urban/industrial emissions (Cu, Pb), and a Tl-specific mixed source. This multi-source apportionment provides a critical scientific basis for implementing targeted, element-specific pollution mitigation strategies in the basin.

### 3.5. Health Risk Assessment

**Figure 8:**
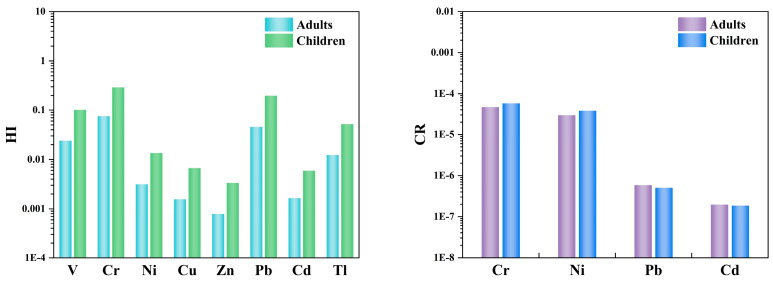
Hazard index (HI) and carcinogenic risk (CR) of PTEs in surface sediments of the Anning River for adults and children exposure groups.

Health risks associated with PTEs in the surface sediments were assessed using the US EPA health risk model (Figure 8, Table S3). Overall, the total non-carcinogenic hazard index (THI) values for adults and children were 1.64 × 10^−1^ and 6.71 × 10^−1^, respectively, both of which were below the safety threshold of 1. This indicates that the studied PTEs are unlikely to pose significant non-carcinogenic health risks to the exposed population. However, the THI value for children was approximately four times higher than that for adults, suggesting that children are more vulnerable to PTE exposure and should be considered the priority group for health protection.

In terms of individual-element contribution, both adults and children showed a similar order of non-carcinogenic risk: Cr > Pb > V > Tl > Ni > Cd > Cu > Zn. Among these elements, Cr exhibited the highest HI values, reaching 7.51 × 10^−2^ for adults and 2.91 × 10^−1^ for children, followed by Pb (4.58 × 10^−2^ for adults and 1.98 × 10^−1^ for children) and V (2.39 × 10^−2^ for adults and 1.01 × 10^−1^ for children). These results suggest that although the overall non-carcinogenic risk is acceptable, Cr, Pb, and V are the dominant contributors, while Tl also deserves attention due to its non-negligible contribution.

Carcinogenic risk assessment showed that only Cr, Ni, Pb, and Cd had calculable carcinogenic risks. The total carcinogenic risk (TCR) values for adults and children were 7.68 × 10^−5^ and 9.60 × 10^−5^ (Table S3), respectively, both within the acceptable risk range of 1 × 10^−6^ to 1 × 10^−4^ recommended by the USEPA. Nevertheless, the TCR value for children approached the upper acceptable limit, indicating a non-negligible potential carcinogenic concern. Among the carcinogenic elements, Cr and Ni were the dominant contributors (Figure 8), with Cr showing the highest CR values (4.65 × 10^−5^ for adults and 5.74 × 10^−5^ for children), followed by Ni (2.95 × 10^−5^ for adults and 3.79 × 10^−5^ for children), whereas the contributions of Pb and Cd were minimal.

Notably, the health risk results were not entirely consistent with the pollution assessment based on the EF, *I_geo_*, and ecological risk indices. Although Cd and Pb were identified as the major pollutants in terms of enrichment and ecological risk, Cr and Ni dominated the carcinogenic health risks. This discrepancy indicates that environmental contamination levels do not necessarily correspond directly to human health risk, which is also governed by toxicological parameters, exposure pathways, and dose–response relationships. Therefore, in addition to controlling Cd- and Pb-related pollution inputs, greater attention must also be paid to the long-term health implications of Cr and Ni in the study area. Overall, the human health risks posed by sediment-associated PTEs in the study area were within acceptable levels; however, children were clearly more susceptible than adults, and special attention should be given to the carcinogenic effects of Cr and Ni, as well as the non-carcinogenic contributions of Pb, V, and Tl. It should be noted that the carcinogenic risks reported herein are screening-level estimates rather than direct representations of actual exposure.

**Figure 9:**
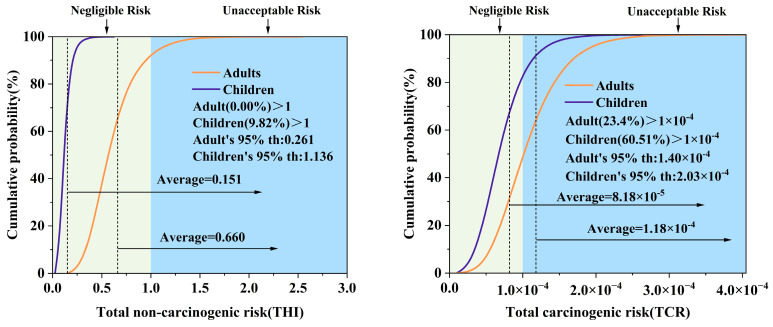
Cumulative probability distributions of THI and TCR for adults and children based on Monte Carlo simulation.

To further quantify the uncertainty associated with human health risk assessment, Monte Carlo simulation (Table S4) was applied to evaluate the probabilistic distributions of total non-carcinogenic risk (THI) and total carcinogenic risk (TCR) (Figure 9, Table S4). The results revealed pronounced differences between adults and children, and the probabilistic assessment provided a more comprehensive characterization of potential health risks than the deterministic approach. For the THI, the simulated mean values for adults and children were 0.151 and 0.660, respectively. The 95th percentile of adult THI was only 0.261, and the probability of exceeding the safety threshold of 1 was 0.00%, indicating that the non-carcinogenic risk for adults was generally acceptable. In contrast, the 95th percentile of children’s THI reached 1.136, and the probability of THI > 1 was 9.82%. These results suggest that although the deterministic THI value for children was below 1, nearly 10% of the simulated exposure scenarios may still lead to unacceptable non-carcinogenic risk when parameter uncertainty is taken into account.

For the total carcinogenic risk (TCR), the simulated mean values for adults and children were 8.18 × 10^−5^ and 1.18 × 10^−4^, respectively. The 95th percentile of adult TCR was 1.40 × 10^−4^, with a 23.4% probability of exceeding the acceptable upper limit of 1.0 × 10^−4^. For children, the 95th percentile reached 2.03 × 10^−4^, and the probability of TCR > 1.0 × 10^−4^ was as high as 60.51%. This indicates that although the deterministic TCR values for both adults and children fell within the acceptable range recommended by the US EPA, the probabilistic assessment revealed a substantial likelihood of unacceptable carcinogenic risk, especially for children.

Overall, the Monte Carlo simulation demonstrated that the non-carcinogenic risk was generally controllable, but children still had a certain probability of experiencing unacceptable risk, whereas the carcinogenic risk was more pronounced, particularly in children. Compared with deterministic assessment, the probabilistic approach captured the upper-tail behavior of risk distributions and showed that point-estimate-based evaluations may underestimate the actual health risk under uncertain exposure conditions. Therefore, children should be considered the priority population for health protection, and greater attention should be paid to the long-term carcinogenic risk associated with sediment-borne PTE exposure.

In both assessment models, children consistently exhibited significantly higher THI and TCR than adults. The physiological and behavioral characteristics of children make them particularly susceptible to sediment-borne PTEs. Their lower body weight results in a higher intake dose per unit of mass. Furthermore, frequent outdoor play, hand-to-mouth activities, and potential pica behavior drastically increase their direct ingestion of contaminated sediments and riparian soils. Given their developing immune and nervous systems, children represent the most vulnerable demographic and require prioritized environmental protection and long-term health monitoring regarding sediment-borne PTE exposure.

### 3.6. Implications for Environmental Management

Mining-induced environmental degradation is a pervasive global challenge, particularly in regions with intensive anthropogenic activities and long histories of mineral exploitation. Place-based scientific evidence plays a role in translating global environmental management strategies into actionable, context-specific practices. This study was conducted in the Anning River Basin, a typical mining-affected watershed in the Panxi region of southwestern China. The findings provide targeted scientific insights for local environmental governance and have broader relevance for mining-impacted regions worldwide. To address the complex pollution profiles observed in this study, differentiated mitigation strategies are required. For Cd and Pb, which show pronounced anthropogenic enrichment and severe ecological risks, management should prioritize stringent source control. Key measures include strictly regulating wastewater discharges from mining operations, upgrading tailings infrastructure to prevent leakage, and treating acid mine drainage at the source. For Cr and Ni, which are primarily lithogenic and difficult to eradicate through source control alone, management efforts should shift toward interrupting exposure pathways.

The protection of vulnerable populations is a consideration. Probabilistic modeling in this study identified a greater than 60% probability of unacceptable carcinogenic risk for children. Restricting children’s access to highly contaminated riparian zones near residential areas is therefore crucial to minimize direct contact with polluted sediments. This finding highlights the importance of incorporating age-specific vulnerability assessments into environmental management policies, particularly in mining communities around the world. Furthermore, seasonal hydrological variations necessitate targeted ecological restoration approaches. During the dry season, exposed sediments are readily resuspended as fugitive dust, creating additional inhalation exposure pathways for nearby residents. Enhancing ecological water replenishment and restoring riparian vegetation represent effective remedial measures to reduce human exposure through both ingestion and inhalation routes.

This study demonstrates how detailed site-specific scientific investigations can strengthen the evidence base for environmental management strategies. The integration of empirical geochemical data with risk assessment frameworks provides a solid foundation for evidence-based decision-making in regions affected by intensive anthropogenic activities. The multi-methodological approach used here, which combines geochemical analysis, ecological risk assessment, and probabilistic health risk modeling, offers a replicable framework for similar mining-affected watersheds.

## 4. Limitations

Several limitations of this study should be noted. The human health risk assessment was performed solely based on total metal concentrations in sediments, without considering metal speciation, mobility, or bioavailable fractions. Since actual human exposure is primarily related to the bioavailable portion of metals rather than their total content, the calculated risks may not fully represent real exposure conditions and could deviate from actual health risks. This is particularly relevant for chromium, whose carcinogenic risk was calculated using total Cr concentrations without speciation analysis. To partially account for the uncertainty associated with metal speciation and exposure parameters, the Monte Carlo simulation incorporated probability distributions, producing probabilistic risk estimates rather than deterministic point values. Additionally, the assessment only considered direct sediment exposure pathways. Other potential exposure routes such as drinking water consumption, dietary intake, and trophic transfer through the food chain were not included, meaning the total health risk from all pathways may be underestimated. Despite these limitations, total concentration-based assessment remains a standard and useful approach for preliminary risk screening. The results provide a conservative baseline for environmental management in the study area. Future studies should include metal speciation, bioavailability analyses, and multi-pathway exposure assessment to refine health risk estimates.

## 5. Conclusions

This study systematically investigated the contamination levels, source apportionment, and probabilistic human health risks of eight PTEs in the surface sediments of the Anning River Basin. Comprehensive pollution and ecological risk assessments identified Cd as the absolute priority ecological pollutant. While V, Cr, and Ni remained largely unpolluted and closely aligned with background levels, Cd exhibited severe enrichment and posed an extreme ecological risk, acting as the overwhelming contributor to the comprehensive potential ecological risk index (RI), followed by the moderate pollution of Pb. The accumulation of these PTEs was governed by a complex interplay of natural and anthropogenic sources. Source apportionment via the PMF model resolved four distinct sources: V, Cr, and Ni were primarily derived from the natural lithogenic weathering of the Panxi vanadium–titanium magnetite belt; Cd and Zn were strongly associated with regional mining and smelting activities; and Pb and Cu originated from mixed traffic and urban sources. Regarding human health, the probabilistic assessment highlighted hidden, unacceptable risks for children. While the deterministic health risks were within acceptable limits, the Monte Carlo simulation revealed that children faced a nearly 10% probability of exceeding the non-carcinogenic safety threshold (THI > 1) and a striking 60.51% probability of exceeding the acceptable carcinogenic limit (TCR > 1.0 × 10^−4^), with Cr and Ni acting as the dominant contributors to the carcinogenic risk.

## Figures and Tables

**Table 1:** Methodological framework and evaluation criteria for PTEs assessments.

**Eq. No**	Formula	Description	Grading Criteria
(1)	*I_geo_* = $\log_{2}\left[\frac{C_{i}}{1.5B_{i}}\right]$	*C_i_* represents the measured concentration of the specific PTE in the sediment sample, and *B_i_* is the background value of the corresponding element in the stream sediments of China [2].	<0, Unpolluted; 0–1, Unpolluted to Moderately polluted; 1–2, Moderately polluted; 2–3, Moderately to heavily polluted; 3–4, Heavily polluted; 4–5, Heavily to Extremely polluted; >5, Extremely polluted; [31]
(2)	$E_{r}^{i}=T_{r}^{i}\times (C_{s}^{i}/C_{n}^{i})$ *RI* = $\sum E_{r}^{i}$	$C_{s}^{i}$ represents the measured value of the PTE in the sediment sample, $C_{n}^{i}$ represents the background value of the corresponding element in the stream sediments of China. $T_{r}^{i}$ for PTEs (V, Cr, Ni, Cu, Zn, Pb, Cd and Tl) are 2, 2, 5, 5, 1, 5, 30 and 10, respectively [32].	$E_{r}^{i}$ < 40, RI < 110, Low; 40 ≤ $E_{r}^{i}$ < 80, 110 ≤ RI < 200, Moderate; 80 ≤ $E_{r}^{i}$ < 160, 200 ≤ RI < 400, Considerable; 160 ≤ $E_{r}^{i}$ < 320, High; 320 > $E_{r}^{i}$, RI ≥ 400, Very high; [33]
(3)	*EF* = $\left(C_{i}/C_{x\;Sample}\right)/\left(C_{m}/C_{x\;Mn}\right)$	*C_i_* represents the concentration of the target PTE in the sediment, and *C_x_* denotes the concentration of the reference element (Mn was selected as the reference element in this study).	1 > *EF*, No enrichment; 1 < *EF* < 3, Minor enrichment; 3 < *EF* < 5, Moderate enrichment; 5 < *EF* < 10, Moderately severe enrichment; 10 < *EF* < 25, Severe enrichment; [34]
(4)	${ADI}_{ing}=C\times {IR}_{ing}\times EF\times ED\times 10^{-6}/BW/AT$ ${ADI}_{derm}=C\times SA\times AF\times ABS\times EF\times ED\times 10^{-6}/BW/AT$ ${ADI}_{inh}=C\times {IR}_{inh}\times EF\times ED/PEF/BW/AT$ $HI=\sum {HQ}_{i}=\sum \left({ADI}_{i}/{RfD}_{i}\right),\;\;CR=\sum {ADI}_{i}\times {SF}_{i}$	*ADI*: average daily intake, *C*: content of PTE, *IR_ing_:* ingestion rate, *EF*: exposure frequency, *ED*: duration of exposure, *BW*: body weight, *AT:* time period, *SA:* exposed skin area, *AF:* skin adherence factor for the sediment, *ABS:* factor of dermal absorption, *IR_inh_*: inhalation rate, *PEF:* particulate emission factor, *RfD:* reference dose for the corresponding exposure route of PTEs, *SF:* slope factor. Relevant parameters are shown in Tables S1 and S2.	/ [32]

## Data Availability

The data presented in this study are available on request from the corresponding author.

## Supplementary Materials

The following supporting information can be downloaded at https://www.mdpi.com/article/10.3390/toxics14070619/s1, Table S1: The health risk parameters for children and adults in the present study. Table S2: Reference dose (RfD, mg/(kg day)) and slope factor (SF, (kg day)/mg) of HMs. Table S3: A summary table of the certainty of THI and TCR in PTEs of deposit sediment. Table S4: Monte Carlo risk assessment parameter values. Text S1: PMF Source Apportionment Modeling Protocol. The references in the Supplementary Materials are listed in the main text [64,65,66,67,68,69,70,71,72,73].
