# Supplementary material for: Contamination, Source Apportionment and Probabilistic Health Risk of Potentially Toxic Elements in Surface Sediments of the Anning River Basin
Source: Toxics. 2026 Jul 15;14(7):619. doi: 10.3390/toxics14070619 (PMC13417149; doi:10.3390/toxics14070619)
Supplement: Supplementary file 1 [file toxics-14-00619-s001.zip › toxics-4345506-supplementary.pdf]

Supplementary Materials

Contamination, Source Apportionment and Probabilistic Health Risk of Potentially Toxic Elements in Surface Sediments of the Anning River Basin

Wenkai Wang [1, †], Pengfei Che [1, †], Jinjin Wang [1, *], Yue Rao [1, 2], Jian Luo [1], Jianbin Chen [1], Junxi Wang [1, 3], Yanchang Kun [4]

[1] School of Environment and Resource, Xichang University, Xichang 615013, China

[2] Institute of Sedimentary Geology, Chengdu University of Technology, Chengdu 610059, China

[3] College of Earth and Environmental Science, Lanzhou University, Lanzhou 730000, China

[4] School of Information Technology, Xichang University, Xichang 615013, China

[†] **These authors contributed equally to this work.**

*Correspondence author:

Jinjin Wang, Xichang University, Xichang 615013, China. E-mail address: jinjin.wangjj@xcc.edu.cn

Table S1. The health risk parameters for children and adults in the present study.

Parameters	Unit	Adult	Children	References
IngR	mg d^{-1}	100	103	Yao et al., 2025; Zhang et al., 2025
InhR	m^3 d^{-1}	20	7.5	Yao et al., 2025; Zhang et al., 2025
ED	a	30	6	Yao et al., 2025; Zhang et al., 2025
EF	d a^{-1}	350	350	Yao et al., 2025
BW	kg	70	16.68	Yao et al., 2025; Ma et al., 2023
AT	d	10950 (NC)<br>25550 (C)	2190 (NC)<br>25550 (C)	Yao et al., 2025; Zhang et al., 2025
SA	cm^2	5700	860	Yao et al., 2025; Liu et al., 2021
AF	mg $(cm^2\ d)^{-1}$	0.07	0.2	Yao et al., 2025
ABS	unitless	0.001 (NC)<br>0.001 (C)	0.001 (NC)<br>0.01 (C)	Yao et al., 2025; Zhang et al., 2025
PEF	m^3 kg^{-1}	1.36E+09	1.36E+09	Yao et al., 2025; Zhang et al., 2025

Table S2. Reference dose (RfD, mg/(kg day)) and slope factor (SF, (kg day)/mg) of HMs.

HMs	RFD_{ing}	RFD_{inh}	RFD_{der}	SF_{ing}	SF_{inh}	SF_{der}	References
Pb	3.50E-03	3.52E-03	5.25E-03	8.50E-03	4.20E-02		
V	7.00E-03	7.00E-03	7.00E-04				
Cr	3.00E-03	2.86E-05	6.00E-05	5.00E-01	4.20E+01	2.00E+01	Al-Rubaye ey al., 2025; Wang et al., 2025
Cu	4.00E-02	4.02E-02	1.20E-02				
Zn	3.00E-01	3.00E-01	6.00E-02				
Ni	2.00E-02	2.06E-02	5.40E-03	9.40E-01	1.70E+00	4.25E+01	
Tl	8.00E-05	8.00E-05	1.00E-05				Liu et al., 2021
Cd	1.00E-03	1.00E-05	1.00E-05	3.08E-01	6.30E+00	3.00E+00	Ahamad et al., 2021

Table S3. A summary table of the certainty of THI and TCR in PTEs of deposit sediment.

HMs	Adult				Children			
	HI	THI	CR	TCR	HI	THI	CR	TCR
V	2.39E-02	1.64E-01		7.68E-05	1.01E-01	6.71E-01		9.60E-05
Cr	7.51E-02		4.65E-05		2.91E-01		5.74E-05	
Ni	3.14E-03		2.95E-05		1.35E-02		3.79E-05	
Cu	1.56E-03				6.69E-03			
Zn	7.87E-04				3.36E-03			
Pb	4.58E-02		5.83E-07		1.98E-01		5.03E-07	
Cd	1.65E-03		1.96E-07		5.91E-03		1.86E-07	
Tl	1.23E-02				5.21E-02			

Table S4. Monte Carlo risk assessment parameter values.

Parameters	Unit	Adult	Children	References
IngR	mg d^{-1}	100 (±1.7)	103 (66, 103, 161)	Yao et al., 2025; Zhang et al., 2025
InhR	m^3 d^{-1}	20 (±1.27)	7.5 (±0.75)	Yao et al., 2025; Zhang et al., 2025
ED	a	30 (±2.74)	6	Yao et al., 2025; Zhang et al., 2025
EF	d a^{-1}	350 (180, 350, 365)	350 (180, 350, 365)	Yao et al., 2025
BW	kg	70 (±10.71)	16.68 (±1.48)	Yao et al., 2025; Ma et al., 2023
SA	cm^2	5700 (±440)	860（430，860，2160）	Yao et al., 2025; Huang et al., 2020:Liu et al., 2021

Text S1 PMF Source Apportionment Modeling Protocol

Source apportionment was performed using the Positive Matrix Factorization (PMF) model implemented in the EPA PMF 5.0 software platform. PMF is a quantitative receptor model based on least squares optimization, originally developed by Paatero et al. The model decomposes the original concentration data matrix into factor contribution and factor profile matrices according to the following equation:

$$X_{ij}=\Sigma_{k=1}^{p}G_{ik}F_{kj}+E_{ij}$$

where X_{ij} is the concentration of chemical species j in sample i; F_{kj} is the concentration of chemical species j in source factor k; G_{ik} is the contribution of source factor k to sample i; E_{ij} is the residual matrix; and p is the number of source factors resolved by the model.

The model seeks to minimize the objective function Q, defined as the ratio of the squared residuals to the input uncertainties:

$$Q=\Sigma_{i=1}^{n}\Sigma_{j=1}^{m}\left(\frac{e_{ij}}{u_{ij}}\right)^2$$

where e_{ij} is the residual for species j in sample i, and u_{ij} is the uncertainty in the measured concentration of species j in sample i. The optimal solution is obtained when Q reaches its minimum value.

The uncertainty matrix was calculated using two approaches depending on the measured concentration relative to the method detection limit (MDL). For concentrations less than or equal to the MDL, the uncertainty was calculated as:

$$u_{ij}=\frac{5}{6}\text{MDL}$$

For concentrations greater than the MDL, the uncertainty was calculated as:

$$u_{ij}=\sqrt{(\sigma\times c)^2+(0.5\text{MDL})^2}$$

where δ is the relative error of the measurement, typically set to an empirical value ranging from 5% to 20%, and c is the measured concentration of the chemical species.

Model performance was evaluated using standard diagnostic criteria including the Q value, residual distribution, and factor interpretability. The optimal number of factors was determined by testing solutions with two to six factors. The four-factor solution was selected because it achieved the best balance between model fit and geochemical interpretability, with a Q(Robust)/Q(True) ratio close to unity and residuals mostly within the range of -3 to 3. Solutions with fewer factors failed to resolve distinct source contributions, while additional factors resulted in split or non-interpretable profiles.

Ahamad, A.; Janardhana Raju, N.; Madhav, S.; Gossel, W.; Ram, P.; Wycisk, P., Potentially toxic elements in soil and road dust around Sonbhadra industrial region, Uttar Pradesh, India: Source apportionment and health risk assessment. Environmental Research **2021**, 202, 111685.

Al-Rubaye, R. F., Kardel, F., & Dehbandi, R. **2025**. Ecological and human health risks of potentially toxic elements (PTEs) in street dust of Al-Hillah City, Iraq using Monte Carlo simulation. *Science of The Total Environment*, ***966***, 178722.

Huang, Y.-N., Dang, F., Li, M., et al. **2020**. Environmental and human health risks from metal exposures nearby a Pb-Zn-Ag mine, China. *Science of The Total Environment*, ***698***, 134326.

Liu, J.; Wang, Y.; Liu, X.; Xu, J., Occurrence and health risks of heavy metals in plastic-shed soils and vegetables across China. Agriculture, Ecosystems & Environment **2021.**

Ma, J., She, Z., Wang, S., et al., **2023**. Health risk assessment of heavy metlas in agricultural soils around the gangue heap of coal mine based on Monte Carlo Simulation. *Environmental Science*, ***44*****(10)**, 5666-5678. Chinese.

Paatero, P.; Tapper, U. Analysis of Different Modes of Factor Analysis as Least Squares Fit Problems. *Chemometrics & Intelligent Laboratory Systems* **1993**, *18*, 183-194.

Wang, P., Han, G., Hu, J., et al. **2024**. Remarkable contamination characteristics, potential hazards and source apportionment of heavy metals in surface dust of kindergartens in a northern megacity of China. *Journal of Hazardous Materials,* ***465***, 133295.

Wang, Z., Lei, K., Lu, X., et al. **2025**. Source-oriented probabilistic risk assessment and priority control factors of potentially harmful elements in fine roadway dust from a representative industrial city, China. *Journal of Environmental Sciences*.

Yao, J., Qian, J., Hu, W. **2025.** Health Risk Assessment of Lead, Zinc, and Cadmium in Soil from a Mining Area in Liuzhou Based on the Bioavailable Forms of Heavy Metals and Monte Carlo Simulation. *Environmental Science*, 1-14. Chinese.

Zhang, Y., Wei, D., Xie, Z., et al. **2025**. Spatial source-oriented analysis and probabilistic health risk assessment of potentially toxic elements in soils integrating the Geo-detector, APCS-MLR, and Monte-Carlo models. *Journal of Environmental Chemical Engineering*, 13(5), 117983.
